# The classroom as a space of resistance. Cooperation, gratitude and collective memory between neuroscience and social science

**DOI:** 10.3389/fsoc.2025.1567675

**Published:** 2025-05-30

**Authors:** Dévrig Mollès, Marcos Parada-Ulloa

**Affiliations:** ^1^Université de Strasbourg, Francia / Programa Sociedad, Cultura y Religión (CEIL-CONICET), Buenos Aires, Argentina; ^2^Instituto IICSE, Universidad de Atacama, Copiapó, Chile

**Keywords:** social science, neuroscience, education, cooperation, gratitude, history, memory, human rights

## Abstract

This article explores social transformation through pedagogy, historical consciousness, social science, and neuroscience. Modern educational systems perpetuate social hierarchies. The meritocratic narrative of neoliberalism is a new form of social Darwinism. Behind this illusion lie the mechanisms of accumulated history, social reproduction, and the inheritance of economic, social, cultural, and symbolic capitals. Collective memory is one of these capitals, cultivated by the elites. In contrast, the memory of the vanquished fades into oblivion. Therefore, democratic pedagogy aims to build collective memory and a historical consciousness of equality, inequality, and human rights. This project requires cognitive and methodological tools for both teachers and students. Our proposal is both theoretical and practical. Theoretically, we aim to build a historical awareness and resilience capacities to address the algorithmic colonization. Cooperative pedagogy and neuroscience bring constructive tools. This approach fosters a new rationalism and complex thinking that unifies natural, social, and human sciences into a cohesive pedagogical praxis. We propose to build collective memory and historical awareness among students, pedagogical team, and families. This involves teacher training, an emotional and prosocial climate, cooperative skills, historical research teams, collecting of family memories, and collective synthesis. The project fosters social bonds and skills for democratic sovereignty. Methodologically, the research employs a critical bibliographical review and content analysis from CAIRN, OpenEdition, ScienceDirect, Web of Science, ERICH+, EBSCO, Scopus, and Google Scholar. The selected bibliography includes authors from Canada, Chile, England, France, Germany, India, Switzerland, the Philippines, and the United States.

## 1 Introduction

Despite the official discourse on meritocracy, numerous studies demonstrate that educational systems reproduce social hierarchies. Class, race, and gender continue to determine individuals' educational performance and opportunities (Bourdieu and Passeron, 1977). In France, for example, the Organization for Economic Co-operation and Development estimates that it would take six generations for a working-class descendant to reach the median income (OCDE, 2020). This determinism concerns even the OECD, a multilateral agency with a neoliberal bent, which acknowledges the fictitious nature of its moderately reformist discourse on “equality of opportunity.”

In fact, neoliberalism—and its meritocratic discourse—is primarily a project to restore class dominance to sectors that saw their fortunes threatened by the rise of social democratic endeavors in the aftermath of World War II (Harvey, 2007). With Giroux (2011), we argue that neoliberal meritocracy is merely a new form of social Darwinism, a variant of the inequality ideology inherited from the 19th century. Based on the logic of the “survival of the fittest,” this perspective has historically served as a justification for class domination and racial colonization. His influence was decisive in Latin America. Its educational models were elitist, denying the public good nature of education, reserving higher education for ruling minorities, and relegating the majority to ignorance (Spencer, 1864a; Blot, 2007).

This creed posits that academic and professional success depends exclusively on individual talent and effort, rendering inherited advantages and structural conditions that affect performance invisible. According to this narrative, individuals deserve (or do not deserve) the rewards that the Market assigns to their talents and efforts, assuming that competition is fair and that all compete under equal conditions. It downplays the weight of “accumulated history” (Bourdieu, 1986, p. 241) —that is, the social trajectory and inherited cultural and symbolic capital— in shaping personal achievements. From a critical perspective, various authors argue that meritocracy does not guarantee genuine equality of opportunity but rather operates under a logic of conditional social mobility: only a small number of individuals manage to ascend the social ladder, and they do so by conforming to the dominant system's values, norms, and demands.

In this framework, mobility is not a structurally accessible possibility for all, but rather an exception that confirms the rule of social reproduction. Most individuals remain confined to their original social positions due to structural barriers obscured by meritocratic discourse. This discourse often holds individuals responsible for their “failure” without acknowledging the historical and social constraints that limit their real chances of advancement. As Sandel (2020) argues, the meritocratic ideal is not grounded in the principle of equality but rather in the demand for mobility. However, this mobility—presented as the desirable horizon of individual progress—is, in practice, profoundly restricted or even blocked, particularly for those from structurally disadvantaged backgrounds (Véron and Ottavi, 2025).

Thus, merit becomes a symbolic trap that legitimizes inequality by presenting an inherently unequal competition as fair. This is the social reality. The religion of the market seeks to transform us into servile and conformist beings (Gori, 2023), while imposing its brutal logic, devastating both society and nature (Harcourt, 2018, p. 5–6). We therefore analyze its psycho-political impact, the rise of cruelty, and a new game-changer: the algorithmic colonization of minds (Benasayag, 2023). This analysis must now be integrated into critical pedagogy. This phenomenon is a key mechanism of the control society described by Foucault and Deleuze. Recent literature in neuroscience and social science underscores this point.

In these conditions, what would be the very foundations of democratic education for 21st Century? We explore cooperative pedagogy and key historical references, integrating neuroscience to find constructive tools in knowledge biology, resilience, and gratitude. This approach fosters a new rationalism and complex thinking that unifies natural, social, and human sciences into a cohesive pedagogical praxis. We propose an applied project to build collective memory and social awareness among students, guided by a pedagogical team. This involves teacher training, creating an emotional climate in the classroom to stimulate pro-social neural circuits and cooperative skills, forming historical research teams to collect family memories, and synthesizing findings collectively. The project connects the educational community, families, generations, and local civil society, fostering skills for democratic sovereignty.

## 2 Method

This study employs a documentary methodology with a bibliographic design and a critical-reflective approach to analyze the importance of cooperation, memory, and consciousness as key components of egalitarian and democratic education in the 21st century. Following Gómez Luna et al. (2014), the literature review identifies and analyzes significant theoretical contributions, facilitating an effective approach to an extensive body of specialized literature. This methodology requires advanced skills in searching, selecting, critically reviewing, and synthesizing relevant academic documents. The corpus was selected through non-probabilistic sampling based on convenience criteria and expert judgment, adhering to guidelines proposed by Muñoz (2013) and Gómez Luna et al. (2014). Databases utilized include CAIRN, OpenEdition, ScienceDirect, Web of Science, ERIC+, EBSCO, Scopus, and Google Scholar. Information was collected using descriptors such as concepts such as “cultural capital,” “critical thinking” and “historical empathy,” “critical consciousness and history,” “human rights,” “social Darwinism,” “competition,” “cooperation,” and “critical education.” These descriptors contribute to the study's central objective and research questions. Practical coding categories included “social history,” “pedagogical praxis,” “society of control,” “algorithmic colonization,” “gratitude,” and “technofeudalism.” The methodology for this critical-reflective documentary review followed the steps proposed by Gómez Luna et al. (2014): defining the problem; searching for information; organizing information; analyzing information. Content analysis, as described by Andréu (2002), was the primary interpretive technique. This technique, focusing on written texts, generates specific knowledge about the study's object. Content analysis was operationalized through a methodological sequence described by Fernández Chaves (2002): identifying analysis topics based on previously established constructs of interest; Collecting data using descriptors from selected databases; Developing coding categories and schemes aligned with the study's objective; Applying the coding scheme to a small sample of compiled material; Coding the entire corpus by applying the coding scheme to all preselected documents; Assessing coding coherence through consistency checks with processed information. On this basis, we propose a first pedagogical project, aware of the need to deepen our analysis and understanding of the challenges of democratic education in the 21st century.

## 3 Critical and historical consciousness

### 3.1 The social world as accumulated history

The social world is accumulated history. Clear and concise, the French sociologist Pierre Bourdieu referred to the formation of social hierarchies as the result of a long historical process. What is accumulated and inherited? Different forms of capital: economic, social, cultural, and symbolic (Bourdieu, 2012). Transmitted from generation to generation, they reproduce asymmetries and inequalities.

The educational system often reflects these inequalities, despite the official meritocratic narrative. How can an alternative pedagogical praxis be developed? Such praxis should reintroduce the notion of capital and all its effects to foster collective critical consciousness among students:

“The social world is accumulated history, and if it is not reduced to a discontinuous series of instantaneous mechanical equilibria between agents treated as interchangeable particles, one must reintroduce the notion of capital and with it, accumulation and all its effects” (Bourdieu, 1986, p. 241).

Collective memory is one of these symbolic capitals. Upper classes cultivate this type of consciousness: typically, for example, the aristocracy maps its lineages and embeds them in the foundation of its collective identity. Subaltern classes, on the other hand, do not map their lineages nor transmit their collective memory, except in politically active families that have accumulated an alternative cultural and social capital. The absence of collective memory is characteristic of the vanquished, as illustrated by immigrants who proliferate around the world (Todd, 2010).

### 3.2 “A totally new history”: equality and human rights

The defeated and subordinates do not cultivate their memory. Building it is a necessary step toward autonomy. To emancipate oneself from the unconscious weight of *accumulated history* and to be strengthened in the process requires the acquisition of social memory. An individual and collective consciousness, inscribed in a past-present continuum, constitutes a strategic objective for a democratic and egalitarian pedagogical praxis.

This strategy also has its own accumulated history. The *Report on Public Instruction* (1792) and *Social Art: On the Admission of Women to the Right of Citizenship* (1790)—both presented to the National Assembly during the French Revolution—is a starting point. These documents establish the need to form not only producers but also active citizens (Mollès, 2020).

In these reports, Condorcet stated that “the teaching of history requires particular attention.” The philosopher and mathematician were not referring to the genealogy of aristocratic lineages and their legitimization, as was then customary. Rather, he spoke of a “totally new history,” conceived not to seduce minds but to shed light—a history of human rights, of equality and social inequality, of consciousness and the enjoyment of these rights:

“But almost all historical literature has been conceived to seduce minds rather than to enlighten them. We therefore need a totally new history, which would be above all the history of human rights, of the vicissitudes to which they have been subjected everywhere, both in consciousness and in the enjoyment of these rights. A history that would measure, according to this criterion, the prosperity and wisdom of nations. A history through which we could trace, in each nation, the progress and decline of equality and social inequality” (Condorcet, 1792 cited in Buisson, 1929, p. 85).

This vision underscores the need for a critical historical consciousness that not only understands the past but also questions the present and envisions the future. It demands an educational approach that challenges the reproduction of social hierarchies and nurtures the collective capacity to envision a more just and egalitarian society.

### 3.3 Seeking an alternative pedagogy

Such a project requires the development of cognitive and methodological tools among both teachers and students. This challenge, undoubtedly, is an experimental, collective, and interactive process, rather than an industrial product that classifies and serially formats passive subjects. It is a maieutic dynamic comparable to artistic creation (Skelton, 2024, p. 353). It transforms all its actors. Teachers and students need self-analysis, theory, and practice to break free from the vicious circle of social reproduction and gain the capacity to “map” (Rabikowska, 2009, p. 238) their position in the world, in time, and in space. Self-awareness and critical reflexivity enable the liberation of the imagination and the construction of an alternative.

In subaltern social contexts, a useful starting point would be to ask: from which accumulated history do we come? This “we” would include teachers, students, educational institutions, and families. It would identify the inherited capitals and the individual and collective determinisms they imposed on their lives, status, behavior, and opportunities.

Renouncing dominant illusions may involve a degree of discomfort: symbolic violence is inevitable in any meaningful process of change. In *The Future of an Illusion* (1927), written about the decline of religious beliefs, Freud acknowledged the inevitability of such discomfort (Harcourt, 2020, p. 203–204). Self-analysis and theory are indispensable, but their outcomes are unpredictable and potentially hazardous for the institution, educators, the community, and students (Rabikowska, 2009, p. 239). The process must be guided by the teacher who, as an intellectual (Giroux, 1988), has the responsibility to foster resilience (Cyrulnik, 2012, 2018) and to liberate the “radical imagination” toward a constructive horizon (Castoriadis, 2008, p. 122–123). To achieve this, it must be anchored in cooperative pedagogies (Casanova et al., 2024) and respond to the “algorithmic colonization” of minds (Benasayag, 2023) from the perspective of the biology of knowledge, love, and gratitude (Maturana Romesín, 2006; Maturana Romesín and Pörksen, 2008).

## 4 Social Darwinism and neoliberal meritocracy

### 4.1 Social Darwinism

In his seminal texts from 1790 to 1792, Condorcet applied the universalist and egalitarian ideology that, since the French Revolution, has provided a legitimacy umbrella for all egalitarian movements. However, the geoculture of the modern world-system was structured based on a dialectic between two “antagonistic and symbiotic” paradigms (Wallerstein, 2005, p. 116–117): universalist-egalitarianism and differentialist-inegalitarianism.

The official gospel of modernity, universalist-egalitarianism, prioritized general rules applied to all individuals who—regardless of their class, race, religion, or gender—were defined by their equality of rights. Principles such as equality before the law or universal suffrage gradually spread as a result of this paradigm. Differentialist-inegalitarianism, in contrast, was its exact opposite. Eager for hierarchies and exclusions, it protected the privileges of dominant elites against the *invasion* of subaltern groups.

Differentialist-inegalitarianism takes various forms. For example, from the 19th century onwards, one of its most robust expressions was social Darwinism—a theory based on the epistemological confusion between evolution and progress (Carr, 2010, p. 179–180; Spencer, 1865). Applying the theory of biological evolution to social sciences led to a serious confusion between evolution (a product of biological legacy) and progress (a product of social acquisition).

During a century of industrialization, urbanization, and colonial expansion, social Darwinism reformulated the old theory of inequality between races, classes, and sexes in modern and scientific-like terms. It made the triumph of the strongest—The Survival of the Fittest—a sociological and historical law. This pseudo-law is the core of the social Darwinist doctrine formulated in the 19th century by Spencer (1864b, p. 444–445, 455–457, 468–469, 474), the main spokesman of this ideology, whose influence on the Latin American elites was profound. Its pedagogical applications tended toward elitist and private schooling. Following Herbert Spencer, it denied the public good nature of education, reserving it for ruling minorities and leaving it to private initiative (Holmes, 1994; Tort, 1996; Blot, 2007).

### 4.2 Neoliberal meritocracy

The parallel with the present is unmistakable. As we approach the end of the first quarter of the 21st century, social Darwinism—and its applications in the field of education—remains vigorous and deeply entrenched. It served as the hidden leitmotif of the conservative restoration launched between 1979 and 1981 by Margaret Thatcher and Ronald Reagan in the United Kingdom and the United States, respectively. It constitutes the core of what we call the neoliberal religion. A religion of brutality and depredation for both the social and natural systems (Harcourt, 2018, p. 5–6).

The pedagogical translation of this worldview is neoliberal meritocracy. Within this mechanical and cold doctrine, actors are “treated like interchangeable particles” (Bourdieu, 1986, p. 241). In this accountant's mindset, within this abstract metric, negative numbers must be eliminated. Positive numbers prevail—namely, the strongest.

In the neoliberal theology, individuals either deserve—or do not deserve—the rewards that the Market spontaneously allocates to their talents and efforts. Neoliberal meritocracy is thus presented as equitable: everyone supposedly has access to the gifts of its invisible hand. The self-made man stands as its defining proof. This illusion is carefully constructed and upheld by the elite. They disseminate, across the connected multitudes, a model embodied by successful competitors. They mythologize individual success stories—along with their material, symbolic, and even erotic rewards.

This belief system obscures the weight of “accumulated history,” of social reproduction, and of the networks of influence that determine access to education, healthcare, transportation, housing, energy, and beyond. A social issue is thus reduced to an individual matter. The mechanisms that produce winners and losers are rendered invisible—for instance, the existence of “dynastic privileges in American universities” (Kahlenberg, 2018).

Nevertheless, this myth inhabits the collective unconscious, where mass ideological apparatuses anchor models, heroes, champions, and ways of being based on *The Survival of the Fittest* theorized in 19th century by social Darwinists intellectuals. This ideology penetrates deeply into the popular classes, even though it is “financially and politically sustained” by small financial and political oligarchies that advocate for “lower taxes and less government redistribution” (Harcourt, 2023, p. 4).

## 5 Psychopolitics and algorithmic colonization

### 5.1 Psycho-political impact

Isolated within the multitude, the subaltern internalizes the symbols and models of social Darwinism. Unable to recognize the weight of “accumulated history” in their own life, they fail to understand that a form of statistical determinism accounts for their inability to *succeed over their rivals*. They remain unaware that “the meritocratic ideal is not based on the principle of equality, but on the demand for mobility” (Sandel, 2021, p. 193), and that in reality, the *social elevator* is blocked (Véron and Ottavi, 2025). This determinism concerns even the OECD (2018), a multilateral agency with a neoliberal bent, which acknowledges the fictitious nature of it's moderately reformist discourse on “equality of opportunity.” The meritocratic game is not only unequal; it is an illusion.

The psycho-political impact of guilt and frustration is devastating. Caught between the meritocratic illusion and the weight of accumulated history, the subaltern begins to fracture. Their efforts are exhausted. Their self-esteem corroded, they sink into anxiety, depression, addiction, lethargy, conformism, and frustration. Subjugated and controlled, they become an agent of the very system that consumes them. Eventually, they may submit to providential figures who offer scapegoats as sacrificial victims, allowing them to discharge their resentment without threatening the established order (Sandel, 2020).

This process of decomposition is already well advanced. Neoliberalism has eroded both social cohesion and the mental health of industrial societies. Propelled by the Anglo-American conservative revolution, the Washington Consensus (1990), the Maastricht Treaty (1993), and the Silicon Valley Consensus (Durand, 2020, p. 16–22), neoliberalism has led to what Emmanuel Todd describes as the *Defeat of the West* (Todd, 2024).

In France, for instance, the democratic and social model inherited from the 1945–1990 period has been dismantled. Deindustrialization has been accompanied by the breakdown of an educational system originally conceived—from the Third Republic and the Popular Front onward—to promote the advancement of the working classes. Recently, for example, the OECD (2018) showed that a descendant of a poor family needs six generations, or 180 years, to reach a median income. The corollary is the intensification of class struggles. Contrary to Marx's predictions, these struggles are more downward than upward: the general trend—encouraged by the mass media—is to vent frustration on those who occupy a lower position in the hierarchy (Todd, 2020).

The United States provides another paradigmatic example. Around the year 2000, it projected an image of invulnerability. Yet, as critical intellectuals had warned (Todd, 2002), its internal decomposition had already begun, rooted in profound demographic, sociological, cultural, economic, and geopolitical forces. Hollywood concealed a racialized, communitarian, differentialist, and unequal modernity, deeply marked by internal divisions (Balibar and Wallerstein, 2018; Itzigsohn, 2013; Itzigsohn and Brown, 2015).

The collapse of historical Protestantism and its replacement by the religion of the market facilitated the drift of its elites into nihilism. The social anomie corroding American society—its moral and intellectual disorientation—has clear markers: rising infant mortality and suicide rates, mass shootings, widespread pornography and addiction, elite corruption, economic precarity, alienation, antagonisms across race, gender, and class… The list goes on Todd (2024).

In the absence of robust systems of social solidarity, a repressive proliferation has emerged. Abandoned to their fate, individuals drift without consistent public morality, without quality public healthcare, transport, or education systems. In contrast, prisons—both private and public—now dominate the landscape, absorbing vast public subsidies while reproducing existing racial and social hierarchies (Bertram, 2025; Harcourt, 2023; Schwellenbach, 2017). Their replacement with systems of prevention, care, and cohesion is nowhere in sight among the current ruling elites. As Bernard Harcourt observes, a *repressive paradigm* prevails—one that is functional to the reproduction of *a particular social order grounded in racial, class, and gender hierarchy*:

“We do not have very good public health or mental health systems, or accessible drug treatment programs. Virtually everything is privatized: the best schools, the luxury treatment centers, the counseling services. The main public institutions for mental health today are jails. The largest psychiatric hospital in the United States is, in fact, the Cook County Jail. We abandon people to their own devices (…) to go into student debt; basically, we leave them alone (…) but the moment they are accused of a crime, then we go after them with everything we've got. We'll spend all the money in the world to punish them (…) A repressive paradigm dominates many liberal democracies and serves to create a particular social order based on racial, class, and gender hierarchy” (Harcourt, 2023, p. 137).

### 5.2 Compulsive competition, cruelty, and sadism

Neoliberalism embraces cruelty and destroys social bonds, states (Giroux, 2025). What is its pedagogical impact? The culture of competition inhibits altruism, empathy, and cooperation, ultimately leading to what has been termed *rivalrous sadism*, according to psychological experiments conducted by Kagan and Madsen (1971, 1972), Madsen (1971), and Madsen and Shapira (1970).

The object of their research was to assess the cooperative abilities of children. Two samples were selected for comparison: children from the United States and Mexico, between the ages of five and ten, from both rural and urban settings. The tests were designed to measure their capacity for social cooperation. The proposed activities required collaborative effort in order to be successfully completed, and success was rewarded.

Anglo-American children demonstrated limited cooperative ability, particularly the older ones, who were more heavily conditioned. They struggled to think cooperatively—even when doing so would have served their own interests. These studies concluded that Anglo-American children were not only irrationally competitive but also frequently displayed sadistic rivalrous behavior: they derived greater satisfaction from the defeat or destruction of the other than from collective success. According to one American pedagogue, this reflects a deep-seated cultural trait:

“Competition is so thoroughly interwoven into American culture and the American way of life that several myths are firmly embedded in our way of thinking” (Kreidler, 1984, p. 67–68).

### 5.3 Algorithmic colonization

This culture is compounded by new devices of biopolitical production, as conceptualized by Foucault (1994, 2009) and further developed by Deleuze (1990) in his reflection on the *society of control*. Since the 17th century, a *disciplinary society* emerged in Western Europe; its power was produced by external institutions that confined subjects, such as schools, prisons, asylums, and poorhouses. The society of control represents the advanced stage of the disciplinary society: it adds technologies that lead subjects to internalize an invisible, omnipresent, and anonymous system of control.

It is the factory of what, at the beginning of the Cold War, Herbert Marcuse called The One-Dimensional Man. American capitalism leads to a closed, totalitarian society that disciplines and integrates all dimensions of existence, both private and public. It is characterized by its ability to assimilate opposition forces into a system they once resisted during earlier stages of capitalism and to systematically manage and mobilize *human instincts* and the *explosive elements* of the unconscious:

“The power of the negative, largely uncontrolled in previous stages of societal development, is dominated and becomes a factor of cohesion and affirmation. Individuals and classes reproduce the repression they experience better than at any previous time, for the integration process occurs, essentially, without overt terror: democracy consolidates domination more firmly than absolutism, and managed freedom and instinctual repression become endlessly renewed sources of productivity” (Preface to the French edition, 1967, in Marcuse, 1993, p. 7).

The society of control is also the society of the spectacle, which accumulates images and symbols reproduced infinitely by institutions and individuals (Debord, 1967). Its pillars are mass media and algorithmic colonization. Marcuse (1993, p. 115) characterized mass media as “the mediation between masters and servants.” Its role in propaganda, psychological mobilization, and the manufacture of consent has been thoroughly analyzed by Chomsky (1990, 2011) and Bourdieu (2016), among others. Fluid and pervasive, they infiltrate the intimate sphere of the family living room. They carry the germ of a *new feudalization of the public sphere*, as Habermas noted in 1962 (Habermas, 2003, p. 70, 172, 203–209). The consumer society was beginning to devour civil society.

Today, this situation is exacerbated by the algorithmic colonization of minds, as noted by Franco-Argentine psychiatrist and psychoanalyst (Benasayag, 2023, 2024b,c). It is the consequence of technofeudalism, a new mode of production that social sciences are beginning to explore (Durand, 2020). Natural and social sciences converge on a critical point: screens are deteriorating our cognitive, reflective, communicative, cooperative, and social capacities. They cloud understanding and atrophy brain functions, reducing them to mere operational tasks, as explained by Desmurget (2021), a neuroscience research director at INSERM.

Youth is at the forefront of this biopolitical battle. Until the 1990s, school socialization was based on face-to-face games involving body, emotion, and reasoning. This was replaced by smartphones and virtual communication. Screens alter brain plasticity and basic functioning, negatively impacting crucial abilities—such as concentration, learning, resilience, and cooperation—while generating addiction, as demonstrated by Haidt (2024), professor of social psychology at New York University. Screens have an addictive power comparable to heroin (Benasayag, 2024a). They foster neurochemical addictions, as evidenced by Lembke (2021), director of research at Stanford University School of Medicine. Industries such as sports, pornography, and gambling exemplify this trend. These educational devices have also infiltrated the school environment.

Addiction and compulsive behavior lead to depression and suicide. Today, depression is the world's primary pandemic. In 2004, the WHO declared it the fourth most significant human disease (Yapko, 2018). Between 2013 and 2018, it rose from second to first place. There are no demographic groups where the curves are declining, summarizes Yapko (2018), clinical psychologist and international expert.

In the educational context, a crucial factor must be highlighted for future teachers: until the age of 25, the brain is still developing. Like malnutrition, addictions leave physiological marks that predispose individuals to chronic disorders, such as depression (Huberman, 2022). The pervasive use of screens in schools not only hinders cognitive development but also undermines emotional wellbeing, creating a generation increasingly vulnerable to mental health crises.

The algorithmic colonization of the mind is thus not merely a technological phenomenon but a profound socio-cultural transformation, reshaping human consciousness, and social interactions. Addressing this challenge requires critically engaging with the social and psychological impacts of digital technologies and advocating for pedagogical approaches that prioritize human connection, critical thinking, and mental health resilience.

## 6 What pedagogical alternative?

Teacher training should, in our view, integrate this diagnosis. It is also essential to establish a horizon: what kind of liberation pedagogy is needed in the 21st century? First, it is crucial to map the long philosophical tradition that can support our praxis. Second, teacher training must include a response to algorithmic colonization, drawing from neuroscience to enhance social skills.

### 6.1 Pedagogies of cooperation

We possess, first and foremost, a rich tradition. This thread of Ariadne establishes a common culture that will guide and evolve through pedagogical congresses and academic publications. Within this tradition, one axis stands out: cooperation. As a natural antidote to rivalrous sadism, it is also a social skill essential for peace, justice, and democracy. To introduce cooperation, we propose starting by assessing the representations of future teachers, for instance, using the test proposed by Kreidler (1984, p. 68). Once certain illusions are deconstructed, the genealogy of cooperativism and its pedagogical applications can be addressed. We do not aim to exhaust this topic here but rather to outline some fundamental hinges.

#### 6.1.1 Cooperation: a classic theme in historical sociology

Durkheim (1922) and Kropotkin (1906) still deserve our full attention, as they responded to the social Darwinism of their time by highlighting the fundamental role of cooperation in human, animal, and plant evolution. For example, in the second edition of “The Division of Labor in Society” (1897), Durkheim presented cooperation as the antidote to social anomie.

In the unregulated capitalism of his era, individuals and groups constantly clashed. The “survival of the fittest” meant the strong crushed the weak. This system could not be stable. Violence and injustice fueled a state of chronic war. It was disingenuous, Durkheim argued, to defend this social order in the name of “individual freedom.” On the contrary, freedom:

“is itself a product of regulation. I can only be free to the extent that others are prevented from using their physical, economic, or other forms of superiority to enslave my freedom; and only social rules can impede such abuses of power” (Durkheim, 1922, p. 3–4).

How could a “moral power” be created to “contain individual egoisms” and “the law of the strongest”? Social cooperation was necessary. Indeed, the starting point could not be the law: neither “the political society as a whole, nor the State” could create a collective conscience, as they were its reflection (Durkheim, 1922, p. 6).

The starting point was civil society, the “natural arbiter to decide between conflicting interests and assign appropriate limits to each” (1922, p. 11–12). In direct contact with social life, civil society could develop common rules and ensure their continuity over time. This moral power was self-instituted within cooperative associations that were socially diverse. Within these associations, a “state of opinion,” a “legal and moral regulation,” and a “sense of duty and justice” emerged (1922, p. 5–6).

Cooperation allowed individuals to share “ideas, interests, feelings, and occupations.” To legitimate material interests, it added “the pleasure of communion, of being one with many,” of “leading a common moral life together.” It was “a source of life (…) that warms hearts, opens them to sympathy, and melts egoisms.” From this emerged the “spirit of sacrifice and selflessness” and the “subordination of particular interests to the general interest” (Durkheim, 1922, p. 15–18).

#### 6.1.2 A long pedagogical tradition

Cooperation has been integrated into the core of various pedagogical practices. Each has its own particularities and comparing them can be enriching for future teachers. Here, we emphasize a common feature: all these practices are based on teams working toward common projects, ultimately materializing in a tangible product—whether artistic, editorial, or otherwise. Emblematic techniques include cooking recipes, murals, newspapers, and theatrical performances. Cooperation, therefore, is a practice (Freinet, 1964).

Cooperation enhances learning and academic performance, as students can emulate, support, and complement each other. In this process, they generate reciprocal bonds, forming integrated, and cohesive communities. It even motivates indifferent or inhibited students, especially those who fear making mistakes. None of these practices follow an *all-or-nothing* approach (Kreidler, 1984, p. 95). Instead, they experimentally strive to avoid individual disengagement by balancing the individual and the collective.

In the context of Latin Europe, we must recall the international *modern school* movement—promoted by Francisco Ferrer in Spain (Morzadec, 2024), Maria Montessori in Italy (Béra and Savoye, 2022; Poussin, 2024), and Élise and Célestin Freinet in France (Peyronie, 2000). All these pedagogical practices share the belief—echoing Durkheim and Piaget (1950, 1978)—that cooperation fosters empathy and the sense of justice.

In Anglo-Saxon Europe, the exiles of the Frankfurt School provided tools to address the fear of freedom (Fromm, 1964) and deconstruct the total fetishism of the commodity as well as “libidinal relationships with commodities, with aggressive motorized artifacts, and with the false supermarket aesthetics” (Marcuse, 1993, p. 8). Adorno and Horkheimer (1944) advocated for an anti-authoritarian education focused on forming active citizens equipped with analytical-critical skills, as well as affective empathy and ethical commitment.

These currents have counterparts in North America. Notably, John Dewey—American philosopher, psychologist, and activist—shared similar views. His leitmotif was democracy as both a way of life and a teaching method (Fabre, 2024). His democratic pedagogy was also grounded in guided experience and cooperative projects (Dewey, 2018a,b). History and geography were central to his approach (Dewey, 2018c). For all these educators, cooperation was the lived, experienced, and collectively constructed democratic culture. It was, in essence, the *democratic method* par excellence (Zask, 2018).

In Latin America, it is impossible to overlook Paulo Freire, one of the most important critical educators of the 20th century, known both for “his theoretical rigor and moral strength” (Giroux, 2010, p. 715). Besides his role in the genesis of critical pedagogy, he developed a highly successful literacy campaign in Brazil before being imprisoned during the military dictatorship established in 1964 and subsequently exiled to Chile and Switzerland. Figura como Pedagogy of the Oppressed (Freire, 1969) was thus an act of resistance, creation, and democratic struggle. Freire rejected the *banking education* model which positions students as passive recipients of vertically imparted knowledge. Instead, he proposed a maieutic approach where the teacher, through questioning, guides students to solve problems and discover concepts.

Education, in this view, is both awareness-raising and empowerment—a political and moral practice that provides knowledge, skills, and cooperative abilities. In this way, students learn to read the world, identify structures of oppression, and envision pathways to transformation. The classroom thus becomes a laboratory for practicing democratic sovereignty. Horizontal and egalitarian dialogue fosters collective reflexivity. For Freire, critical history was particularly significant, as it served to understand the present, rise above immediate experience, and imagine possible futures (Giroux, 2010). In essence, it represents a pedagogy of *radical democracy* (Dubigeon and Pereira, 2024).

### 6.2 Critique and counter-critique: a contemporary debate

Theoretical and practical research remains active in critical pedagogy, and teacher training must, therefore, engage with its most recent developments, particularly the critical and counter-critical pedagogies formulated by Henry Giroux, Bernard Harcourt, and Kevin Skelton.

#### 6.2.1 Critical pedagogy

Henry Giroux, from McMaster University (Canada), developed the concept of critical—or radical—pedagogy, drawing from Paulo Freire's work (of whom he was a disciple), combined with the heterodox Marxism of Gramsci and the Frankfurt School, among others (Giroux, 1986, p. 12). As a critic of the school system that, under the guise of meritocracy, reproduces social hierarchies, Giroux envisions critical education as a possibility for the emancipation of subaltern groups (classes, races, and genders). Gramsci's influence is evident in his conception of the teacher as an intellectual tasked with waging this egalitarian and democratic cultural battle (Giroux, 1988).

Like his European and American predecessors, Giroux places significant emphasis on education in public democratic life. Consciousness, autonomy, social justice, equality, solidarity, and the common good lie at the heart of his model, conceived as an intersection between education, culture, and democratic sovereignty (Giroux, 2021, p. 5). Students develop critical consciousness and prepare to transform society. This implies changing pedagogical practices, breaking away from authoritarianism, and prioritizing egalitarian cooperation (Giroux, 2001).

In the 21st century, this educational model transforms the school into a democratic and egalitarian bastion. As transnational corporations capture public space (Dowbor, 2016), a liberal-fascist hybrid emerges (Giroux, 2024). Subjected to the functionalist mandates of the market, education becomes devoid of any vestige of critical thought, reflexivity, and imagination. Conservative ideologies and many teachers or administrators “lack a broad vision or a critical understanding of education as a force for developing imagination and public democratic life” (Giroux, 2010, p. 715). Under siege, egalitarians and democrats find in liberation pedagogies a stronghold of resistance.

#### 6.2.2 Counter-critical education

Formulated in dialogue with critical pedagogy, the counter-critical theory seeks to respond to a context of social and ecological devastation, attacks on intellectuals and scientists, and the rise of oppressive political regimes linked to multinational corporations in finance, energy, information technology, and the commercialization of all forms of life. Bernard E. Harcourt, from Columbia University, observes the fragmentation of democratic counter-powers. He seeks to unify them by proposing autonomy as a horizon (Harcourt, 2018, p. 5–6). In this sense, he aligns with a tradition marked by self-managed social centers in Italy, Spain, France, and Germany during the 1970s−1990s, the indigenous Zapatista resistance caracoles in Chiapas from 1991 onwards, and the French ecological movements of recent decades (some referenced by Harcourt, 2020, p. 393–394).

Counter-critical pedagogy “encourages diverse modes of knowledge and holistic approaches to community building—concepts predominantly drawn from indigenous philosophy and artistic research,” as noted by the Swiss scholar Skelton (2024, p. 348). It advocates for the development of artistic expression, removing the primacy of verbal and written language: “what we put into words will always be less than what we are trying to describe,” particularly in a context of cultural diversity (Skelton, 2024, p. 357–358). It is a pedagogy of processes, meant to be embodied in concrete and real situations. This approach aligns with the cooperative tradition, grounded in:

“democratic participation, equity, solidarity, respect for others and the environment (…) equitable distribution of wealth, care for social well-being and the Earth, sustainability, mutual aid (…) from consumer cooperatives to credit cooperatives, from workers' cooperatives to mutual aid societies (…) There is no need to wait to realize this ambition. There is no need to convince the majority of other people. There is no need to take power or dismantle the state (…) Models of cooperation are within reach. The basic values and principles are as clear as crystal. We can do it together now” (Harcourt, 2023, p. 199).

### 6.3 The biology of knowledge, resilience, and gratitude

Finally, educators and students must confront algorithmic colonization. It seems, therefore, indispensable to integrate complex thinking into their education, bridging natural sciences, social sciences, and humanities. In the words of the French sociologist and educator Edgar Morin:

“The humanities and social sciences are not aware of the physical and biological characteristics of human phenomena. The natural sciences are not aware of their inscription within a culture, a society, a history. Science is not aware of its role in society. Sciences are not aware of the hidden principles that order their clarification. Science does not know that it lacks consciousness. But from all sides arises the need for a science with consciousness. It is time to become aware of the complexity of all reality—physical, biological, human, social, political—and of the reality of complexity. It is time to become aware that a science devoid of reflection and a purely speculative philosophy are insufficient. Consciousness without science and science without consciousness are mutilated and mutilating” (Morin, 2017).

Recent research has demonstrated what practices like yoga and religions have cultivated for centuries: practice forges communities, improves social relationships, and enhances consciousness, happiness, and resilience (Arleo, 2024; Bono and Duffy, 2023).

Gratitude has effects as potent as exercise or pharmaceuticals, for example, in its ability to reduce TNF-alpha and other inflammatory markers (Hazlett et al., 2021). Regular practice has a lasting impact on emotional, social, and cognitive capacities because it directly affects neural circuits. It involves two antagonistic neural circuits: the defensive circuit and the pro-social circuit.

Each reacts and manifests differently in experiments, involving distinct areas of the anterior singular cortex and the medial prefrontal cortex, regions associated with planning, deep thinking, contextualization, and interpretation of sensory experience. The defensive circuit involves neurochemicals like epinephrine, adrenaline, and cortisol; it generates reactions of fear, protection, flight, aggression, and is dominant due to our evolutionary history. The pro-social circuit involves dopamine, serotonin, acetylcholine, and oxytocin; it favors empathetic and pro-social thoughts and behaviors (Fox et al., 2015).

Gratitude is a pro-social mental state that can be easily trained. Robust studies show that repeated and effective practice activates the pro-social circuit and neutralizes the defensive circuit (Hazlett et al., 2021; Kyeong et al., 2017). It reorganizes our nervous system and leads to a calm, receptive, empathetic, and cooperative mode of being (Kemper, 2025).

## 7 A pedagogical proposal

### 7.1 From the concrete to the universal

How can the classroom be transformed into a space for social cooperation among students? How can this virtuous energy be extended to their families and communities? How can a critical awareness of the *accumulated history* that weighs upon them like a glass ceiling be forged? How can their capacity to overcome such determinisms be strengthened^1^? How can their awareness of human rights, equality, and social inequality be nurtured? How can their autonomy and democratic sovereignty be elevated? These are the aims of this (multi)annual pedagogical project, which must fundamentally be understood as a flexible, variable, and mutable process.

One possible approach begins with the concrete-universal: to move, from one situation to another, toward a form of *sentipensamiento*—a feeling-thinking awareness of self in time and space, an awareness that is at once individual and collective, embodied, symbolized, and conceptualized (Debord, 1967). From lived experience—felt through the body, the senses, and emotions—to analyzed experience, articulated through words and signs. It involves traversing multiple, relative, and interdependent temporalities: personal memories, collective memory, and between the local and the global, glocal history.

The task is to map this terrain. To compare its contours, accidents, and horizons. To recognize similarities and differences in collective experience and translate them into visual symbols that becomes anchored in collective consciousness. Gradually, a map emerges. The territory is organized. Symbols give rise to concepts—concepts that help structure territory, experience, and time. Concepts that draw learners closer to accumulated history from the perspective of the present and lived experience.

### 7.2 Phases

This project comprises four phases. The first involves the training of teachers based on the diagnosis and alternatives outlined in the first two parts of this text. The second phase focuses on developing resilience and cooperative capacities in both students and teachers. The third phase establishes small groups tasked with investigating the collective memory of their families. The fourth phase brings these groups together to compare their trajectories, symbolize them, map them, and translate them into concepts appropriate to each age group.

This project would enhance ties between the educational community, families, generations, and local civil society. It would crystallize a historical consciousness and latent critical-reflexive capacities necessary for the exercise of democratic sovereignty. Indeed, democracy presupposes, as Cornelius Castoriadis reminds us, that “all its members” must develop and exercise throughout their lives the capacity to “participate in reflective and deliberative activities”:

“Democracy, in the full sense of the word, can be defined as the regime of collective reflexivity […] democracy cannot exist without democratic individuals, and vice versa” (Castoriadis, 2008, p. 122–123).

### 7.3 The emotional climate

Before engaging in cooperative projects—or even before acquiring cooperative skills—it is necessary to establish a particular climate. To this end, we suggest building a sense of community, stimulating pro-social neural circuits through effective practices of gratitude, and then developing cooperative skills through the theory of cooperative games, implementing cooperative learning strategies, and finally building and advancing a concrete project.

#### 7.3.1 Emotions and intelligence

In the words of Chilean biologist and educator Humberto Maturana (cita), “different emotions have different effects on intelligence; thus, envy, competition, ambition […] diminish intelligence; only love enhances it”:

“Different emotions have different effects on intelligence; thus, envy, competition, ambition […] reduce intelligence; only love enhances intelligence. Therefore, for the educational space to be one of expanded intelligence and creativity, there can be no evaluations of students' being, only of their doing (…) The separation between science and philosophy is the result of an artificial classification. This separation between thought and action limits our understanding of what we do as human beings in our real lives. It impairs our understanding of the different worlds we generate in our lives (…)” (cit. in Campos Pellanda, 2013, p. 3–5).

The foundational tenets of the new rationalism and of complex thought have, of course, been confirmed by recent research—for example, by Immordino-Yang et al. (2024), Immordino-Yang and Fischer (2010), and Immordino-Yang and Yang (2017), professor of education, psychology, and neuroscience at the University of Southern California, and director of the Center for Affective Neuroscience, Development, Learning, and Education. Her research on emotions, self-awareness, and social interactions has confirmed their impact on our cognitive and interpretative capacities. Emotion conditions reason, in both children and adults. Therefore, she calls for a comprehensive reform of the educational system to include new modes of exploration and multiple forms of learning.

#### 7.3.2 Gratitude practices

In response to the algorithmic colonization of human minds and the social entropy that ensues, we propose the science of gratitude. Disciplines such as yoga and pranayama—which could provide an excellent foundation for non-competitive physical education—have cultivated the art of gratitude for millennia. Today, both the natural and social sciences confirm that regular gratitude practice enhances physical, mental, and social wellbeing. It fosters resilience against past and future traumas, increases happiness and cognitive performance, and liberates both intuition and reason, as well as emotional and rational intelligence (Arleo, 2024; Bono and Duffy, 2023).

Gratitude practices can be integrated into educational contexts to improve the wellbeing of both students and educators. They can strengthen resilience in the face of academic and personal challenges, promote positive social relationships, and foster a cooperative environment. Moreover, these practices can be adapted to diverse cultural settings with demonstrably positive outcomes (Garg, 2020; Hameed and Khwaja, 2023; Howells, 2014; Liauw, 2019; Sumari et al., 2025; Valdez et al., 2022).

The most powerful form of gratitude practice is receiving recognition for altruistic acts one has performed for others. An effective practice should be repeated regularly, ideally two to three times per week. Holding one main session and several brief micro-sessions of a few minutes—supported by prompts or symbolic reminders—has proven to be effective (Chauhan et al., 2025; Valdez et al., 2022).

Gratitude practices should be anchored in narrative (Cyrulnik, 2023), whose authenticity is essential in order to provoke genuine social resonance. Narratives possess the power to synchronize brain, heart, and body. Recent experiments have confirmed this: they demonstrate that the heart rates of disconnected individuals can align when they are exposed to the same narrative (Fox et al., 2015; Pérez et al., 2021).

Effective practices, therefore, are rooted in believable stories that enable emotional transference, empathy, and portray individuals receiving help. This type of narrative structure activates prosocial neural circuits more effectively than other forms of practice. One pedagogical approach involves drawing upon local and universal cultural heritage to structure regular gratitude practices. The study of certain songs can serve as a powerful support, as exemplified by *Chanson pour l'Auvergnat* and *La Mauvaise Réputation* by French singer-songwriter Brassens (1954), the latter famously interpreted in Spanish by Paco Ibañez (Brassens and Ibañez, 2008a,b).

### 7.4 Cooperative skills

Fostering a psycho-emotional climate of this nature provides the foundation for the acquisition of cooperative skills through games and activities designed to build trust, encourage teamwork, and promote the sharing of roles and knowledge. Both research and practice have demonstrated the superiority of cooperative groups in creative problem-solving, for instance. Learning to cooperate is foundational:

“Of all the aspects of a peaceful classroom, cooperation is in many ways the most important. Creative conflict resolution, tolerant attitudes, good communication habits, and the appropriate ways to share feelings are all much more likely when children know how to work together. Cooperation also builds a sense of community and well-being” (Kreidler, 1984, p. 66).

#### 7.4.1 Cooperative routines

In addition to gratitude, it is both possible and desirable to implement other routine-based practices. Among these, we may highlight modes of conflict resolution, a subject detailed by Kreidler (1984), who provides numerous practical examples. Other crucial elements include the incentivization of virtuous behavior and the implementation of cooperative learning strategies. Establishing routines and procedures that encourage and reward cooperation constitutes a powerful pedagogical tool.

Physical contact, in this context, is particularly valuable. For instance, according to Kreidler (1984), in China, children traditionally button their clothing at the back and must help one another. Certain tedious tasks can become learning opportunities. For example, untied shoelaces can give rise to cooperation and gratitude between children who do not yet know how to tie them and those who do.

Another virtuous routine is to praise and reward cooperative behavior. Virtuous acts can be publicly acknowledged through oral celebration. Collectively recounting these episodes—at the end of the week, for example—can be integrated into gratitude practices. The effect is enhanced when students are invited, on a rotating basis, to create and share these lists of commendable actions.

#### 7.4.2 Games and activities

Game is one of the fundamental forms of socialization across all species, used from early childhood as a medium for learning and social integration. This aspect of play is widely acknowledged in the sociology of education and sport, where its pedagogical applications have been extensively studied (Callède, 2010; Kapp, 2015; Le Breton, 2010).

In the field of game theory—particularly as developed during the 1990s—scholars explored how players can learn to cooperate through repeated interaction and the adaptation of strategies, ultimately arriving at stable outcomes such as *Nash equilibria*. Evolutionary theory suggests that over time, and with accumulated experience, individuals increase their capacity for cooperation. Despite these insights, neoliberal orthodoxy has favored the study and promotion of competitive games, especially within the teaching of economic theory. This reflects an ideological stance that underestimates the power of cooperation to resolve social and economic challenges. However, some studies have shown that cooperative games can lead to advantageous equilibria and help mitigate the disadvantages of excessive competition and the concentration of power. This alternative perspective offers significant transformative potential—provided it receives the attention it merits (Harcourt, 2023, p. 90–92).

The disinterest of neoliberal economists contrasts sharply with real-world experience. In schools, cooperative games offer a relaxed and informal opportunity for growth. Kreidler (1984, p. 72–80) cites numerous practical examples and identifies four methodological principles for such games: simultaneous ending (where all players complete the final move together), coordination of timing and movement (to generate collective fluidity), rotation (where each player takes a turn in sequence and contributes an indispensable step toward the final goal), and a predetermined score that requires players to combine efforts to achieve it.

Cooperative activities serve as a complementary strategy. These are process-oriented exercises unrelated to academic subjects, devoid of performance pressure, and focused on imagination and social-emotional skill development. Story invention, for example, can stimulate creativity—as exemplified in The Rat's Manifesto (Tasso, 2025).

#### 7.4.3 Teams

To teach cooperation, Kreidler (1984, p. 66, 71, 80) emphasizes the importance of groups. Whether physical or conceptual, these groups are unified by a common purpose. Instruction in cooperative skills begins with the formation of pairs, then trios, groups of four, and so on, increasing in size as the school year progresses and as students' competencies develop. He stresses that learning to cooperate takes time.

To avoid stagnation, groups should be regularly mixed. The teacher plays a background role, supporting students as they navigate conflicts and highlighting the constructive potential of disagreement. They provide encouragement and patience, observing the strengths and weaknesses of group dynamics and addressing these later during reflective discussions.

In the face of challenges, the solution is not to provide answers, but to encourage cognitive processes. Providing immediate answers relegates students to the role of passive recipients and burdens the teacher. In this framework, it is highly recommended to establish a team rule whereby students must seek help from all group members before turning to the teacher.

Implementing a peer tutoring system offers significant benefits (Peyrat, 2009). Tutors serve on a rotating basis to prevent the establishment of asymmetrical roles. They are designated to share their expertise in specific tasks. Their mission requires a degree of training: their goal is not to provide answers, but to assist others in discovering them. Experience has demonstrated the effectiveness of this method.

Interlinked (or jigsaw) groups represent the culmination of this process. This method was developed by psychologist Elliot Aronson and his colleagues. Ideally composed of five students, these groups focus on a specific topic. A practical example might be the local municipality: its political organization, public services, population origins, and socio-economic composition, among other aspects. The teacher provides the study materials, dividing them thematically and assigning one topic to each student. Nevertheless, all students are responsible for mastering the entirety of the material. This means they must work collaboratively, teach one another what they have learned, ask questions, and support each other. In the final stage, an individual assessment is conducted. Though this method demands a high level of cooperative skill, it has proven to be a source of both academic and interpersonal progress (Kreidler, 1984, p. 102).

### 7.5 Collective memory and social consciousness

This project culminates in a research initiative centered on the collective memory of students' families (Duby, 1997). The outcomes of the various working groups are integrated into a visual and symbolic synthesis, which in turn serves as a foundation for the acquisition of age-appropriate conceptual knowledge.

The project's objectives aim to consolidate the cooperative skills previously acquired while fostering in students a deeper awareness of their social, historical, and geographical position in the world. In abstract terms, the goal is to instill principles of analytical and critical thinking, as well as a meaningful and enduring individual and collective memory imbued with symbolic significance.

The project is structured around the construction of a collective memory, with students' families serving as the primary protagonists. The documentary basis consists of oral and visual testimonies, which are analyzed by the student working groups and later synthesized in a comparative phase. The outcome of this stage takes the form of a community genealogical display. In addition to a simplified genealogical chart, the display incorporates visual symbols representing ethnic, migratory, and socioeconomic origins, inherited forms of capital (economic, social, and cultural), and other relevant aspects that emerge throughout the process.

This collective discovery will serve to anchor a core of shared memory and consciousness. Through this exercise, essential critical concepts for the analysis of modern industrial society will be crystallized, enabling students to better understand their place in the world, their origins, resources, and opportunities. Furthermore, the project seeks to cultivate historical empathy and promote a spirit of benevolence among those who have inherited more, toward those with fewer resources.

#### 7.5.1 Phase 1: introduction to historical research

The initial phase takes place in the classroom and consists of introducing students to the process of historical inquiry. Students are divided into groups, and each group is assigned a specific space-time context to investigate. This may pertain to the local territory, the regions of origin of students' families, or entirely unfamiliar (even exotic) contexts. The objective is to identify practices of cooperation within these environments. Once these findings are presented, a group discussion follows: How were these examples identified? Which examples are similar to current ones? Which differ? Are some examples more or less cooperative than others?

#### 7.5.2 Phase 2: guided collection of social memory

In this phase, the research groups conduct fieldwork, following parental consent. A questionnaire—designed by the participating teachers—is provided, aimed at constructing family narratives. The objective is to strengthen social bonds, stimulate self-awareness, and reinforce collective identity. The questionnaire includes sections on family genealogy, encompassing ethnic, migratory, and labor backgrounds; accumulated forms of cultural, social, and economic capital; access to public services (health, education, energy, and transport); experiences of cooperation and solidarity within the family, neighborhood, and workplace; and experiences of civic mobilization.

#### 7.5.3 Phase 3: collective synthesis and genealogical display

In this phase, the different research groups come together to develop a synthesis, ideally materialized in the form of a community genealogical display enriched with visual symbols corresponding to previously introduced critical concepts. Kreidler (1984) offers various procedural models—such as group storytelling—that can serve as useful references. Each group initially prepares its individual contribution, and then, in a second step, the combined groups organize the collective display using shared symbolic codes.

#### 7.5.4 Phase 4: collective reflexivity

Once the community display is completed and its cooperative achievement celebrated, the project enters a phase of collective reflection and deliberation. This phase can be divided into two segments: small cooperative problem-solving groups and, subsequently, interlinked (jigsaw) groups. A training component on cooperative problem-solving may be included, following the model proposed by Kreidler (1984), based on real-life situations, and aimed at encouraging democratic participation and the search for practical measures to improve the social, economic, and cultural conditions of these families. The teacher must assess whether the class has attained the necessary level of maturity for such engagement.

## 8 Discussion and conclusions

This research critically examines the impact of the neoliberal paradigm on contemporary education, focusing on neoliberal meritocracy and its roots in modern social Darwinism. From a sociocritical perspective, it demonstrates that the current meritocratic discourse perpetuates social inequalities under the guise of fair competition and individual effort.

Theoretical analysis reveals that neoliberal meritocracy legitimizes individual success without considering structural conditions, shifting the responsibility for failure onto individuals and obscuring the dynamics of privilege and power. This logic is exemplified by the myth of the self-made man, which ignores historical and inherited inequalities that constrain social mobility.

The study also explores algorithmic colonization and its impact on pedagogical practices. Technological dependence and digitalization introduce new forms of control and discipline, altering cognitive and affective processes, and challenging the formation of critical and autonomous subjectivities.

In response, the proposed pedagogical project aims to build a pedagogy of cooperation and critical historical thinking, drawing on democratic and emancipatory educational traditions. Cooperative methodologies counteract the social fragmentation fostered by neoliberal individualism, promoting essential social skills for democratic citizenship. We advocate for transforming the classroom into a space of resilience and resistance where collective memory and critical awareness can flourish.

The pedagogical model departs from neoliberal meritocratic logic, advocating for education oriented toward cooperation, critical thinking, and historical awareness. Emphasizing collective memory and strengthening the educational community fosters more inclusive and democratic pedagogical practices.

The sociocritical approach highlights the need to transform the classroom into a space of critique and creation, where students acquire tools to challenge the existing social order and build alternatives grounded in social justice and solidarity. Consolidating critical historical thinking is essential to denaturalize hegemonic narratives that legitimize inequality, promoting an educational praxis committed to social transformation.

This pedagogical proposal seeks to strengthen critical and active citizenship, enabling collective reflection and action to overcome structural barriers that limit the full exercise of rights. Education thus becomes an effective means of promoting equity and social justice, fostering practices that challenge the status quo and pave the way toward a more democratic and equitable society.

## Data Availability

The original contributions presented in the study are included in the article/supplementary material, further inquiries can be directed to the corresponding author.

## Footnotes

^1^“For the child, 'understanding time means freeing oneself from the present”' (Piaget): Le Goff (1990, p. 9).
